# Towards a better understanding of *Fagopyrum dibotrys*: a systematic review

**DOI:** 10.1186/s13020-021-00498-z

**Published:** 2021-09-16

**Authors:** Le-Le Zhang, Yan He, Feiya Sheng, Ying-Fan Hu, Yu Song, Wei Li, Jiarong Chen, Jinming Zhang, Liang Zou

**Affiliations:** 1 School of Basic Medical Sciences, Chengdu University, Chengdu, China; 2 Key Laboratory of Coarse Cereal Processing of Ministry of Agriculture and Rural Affairs, Chengdu University, Chengdu, China; 3 Affiliated Hospital of Chengdu University, Chengdu, China; 4 College of Pharmacy, Chengdu University of Traditional Chinese Medicine, Chengdu, China

**Keywords:** *Fagopyrum dibotrys*, Phytochemistry, Pharmacology, Functional genes, Active ingredients

## Abstract

*Fagopyrum dibotrys* (*F. dibotrys*) (D.Don) H.Hara is a well-known edible herbal medicine in Asian countries. It has been widely used for the treatment of lung diseases, swelling, etc., and is also an important part of many Chinese medicine prescriptions. At present, more than 100 compounds have been isolated and identified from *F. dibotrys*, and these compounds can be primarily divided into flavonoids, phenols, terpenes, steroids, and fatty acids. Flavonoids and phenolic compounds are considered to be the main active ingredients of *F. dibotrys*. Previous pharmacological studies have shown that *F. dibotrys* possesses anti-inflammatory, anti-cancer, anti-oxidant, anti-bacterial, and anti-diabetic activities. Additional studies on functional genes have led to a better understanding of the metabolic pathways and regulatory factors related with the flavonoid active ingredients in *F. dibotrys*. In this paper, we systemically reviewed the research advances on the phytochemistry and pharmacology of *F. dibotrys*, as well as the functional genes related to the synthesis of active ingredients, aiming to promote the development and utilization of *F. dibotrys*.

## Introduction

*Fagopyrum dibotrys* (*F. dibotrys*) (D.Don) H.Hara, also called *Fagopyrum acutatum* (Lehm.) Mansf. ex K.Hammer, is a perennial herb of the dicotyledonaceae family Polygonaceae [1]. *F. dibotrys* is widely distributed across the North temperate zone and is mainly grown in China, India, Thailand, and Vietnam. China is documented to be the main distribution region of *F. dibotrys*, and wild *F. dibotrys* has been listed as a national second-level protected plant [2]. It has been approved as a functional food by the National Health and Family Planning Commission of the People’s Republic of China [3]. The grains of *F. dibotrys* are common food product used to make side dishes, flour, and tea, and its stem and leaf are used as forage feed resources.

In addition to the nutritional and economic values, the rhizome of *F. dibotrys* is also used as a traditional Chinese medicine for a long history [3, 4]. According to the Pharmacopoeia of China (2020 edition), the rhizome of *F. dibotrys* possesses the effects of clearing away heat and detoxification, expelling pus and removing blood stasis, and is recommended for diseases such as lung abscesses, measles pneumonia, swelling and pain in throat and dysmenorrhea, inflammation, and so on. It is also an important part of many famous traditional Chinese medicine prescriptions such as Wei Mai Ning Capsule and Ji Zhi Tang Jiang.

In the past decades, phytochemical researches on *F. dibotrys* have identified more than 100 compounds, mainly including flavonoids, phenolics, triterpenoids and tannins, supporting its potential to be developed as a health care product and functional food [5]. Pharmacological studies have revealed that *F. dibotrys* possesses anti-inflammatory, anti-cancer, anti-oxidant, anti-bacterial, and anti-diabetic activities. The main bioactive components related with these pharmacological activities have been investigated, as well as the underlying mechanisms. Besides, with the developments in molecular biology technology, research into the functional genes helps to elucidate the biosynthetic pathways and regulatory mechanisms of active ingredients in medicinal plants. Additional studies on functional genes have led to a better understanding of the metabolic pathways and regulatory factors related with the active ingredients in *F. dibotrys*.

This article reviews research progress regarding the chemical compositions, pharmacological activities, and functional genes of *F. dibotrys*, aiming to provide critical information for further research on *F. dibotrys*.

## *Fagopyrum* species

*Fagopyrum dibotrys* belongs to the genus *Fagopyrum* (family Polygonaceae) which comprises more than ten species of plants. Among the *Fagopyrum* species, *Fagopyrum esculentum* (*F. esculentum*) (common buckwheat), *Fagopyrum tataricum* (*F. tataricum*) (tartary buckwheat), and *F. dibotrys* (golden buckwheat) attract increasing attention owing to their long history of both edible and medicinal uses [1]. These three *Fagopyrum* species share similarities in nutritional and medicinal values, whereas there are also many differences between them.

*Fagopyrum esculentum* and *F. tataricum* are annual herbs with small white or pinkish flowers and edible triangular seeds, while the seeds of *F. tataricum* are smaller than that of *F. esculentum*. *F. esculentum* and *F. tataricum* are extensively produced and consumed as the main buckwheats worldwide with more than 4000 years cultivation [6]. Compared with *F. tataricum*, the seed of *F. esculentum* has advantages of sweet taste, large size, and easy dehulling of seed coat. However, detailed comparison studies indicated that the nutritional value of *F. tataricum* is much higher than *F. esculentum*. *F. tataricum* is richer in nutrients and antioxidants, such as vitamin B and rutin, and has good medicinal potential [7, 8]. The antioxidant potential, total phenolic content, and total flavonoid content of *F. tataricum* are much higher compared to *F. esculentum* [6]. In contrast, *F. dibotrys* is a perennial wild herb with the characteristics of a thick main root, whorls, and large achenes. Similarly, the seeds of *F. dibotrys* are also edible, and its stem and leaf are also documented to be good forage feed resources [3]. Its leaves are rich in rutin, making it a healthy addition to diet, and can be boiled or steamed like spinach.

The existing methods to distinguish the buckwheat varieties mainly include morphology, allozyme variability, restriction fragment length polymorphism of chloroplast DNA, rbcL-accD region nucleotide sequence of chloroplast DNA, and nucleotide sequences of the ITS region of rRNA gene, etc. [9, 10]. The use of chloroplast DNA sequence data plays an important role in illuminating the phylogeny of buckwheat species. Previously, the chloroplast DNA sequence of *F. dibotrys* has been well investigated, and the chloroplast DNA size is detected to be 159,320 bp. Compared with the chloroplast DNA of other buckwheat species, *F. dibotrys* was more conserved with several variation hotspots [2]. Wang et al. [11] analyzed the complete chloroplast DNA of two cultivated buckwheat *F. tataricum* and *F. esculentum* together with two wild type *F. dibotrys* and *F. luojishanense*. Their results indicated that *F. dibotrys* is more closely related to *Fagopyrum tataricum*. By determining the DNA sequences of rbcL and accd59 coding regions and their intergenic regions in multiple taxa of *Fagopyrum*, it was found that the evolution speed of accd59 coding region and intergenic region were about 5 times that of the rbcL coding region. *F. dibotrys* has multiple polyploids, and *F. dibotrys* and *F. tataricum* are closely related [12]. However, owing to the incomplete sampling, insufficient chromosome and phylogenetic markers, and complex evolutionary issues, the phylogenetic relationships, metabolism and genome comparisons among the *Fagopyrum* species are still not fully understood, and much more work are needed.

## Ethnomedicinal uses of *F. dibotrys*

Compared with *F. esculentum* and *F. tataricum*, *F. dibotrys* is more famous for its ethnomedicinal uses. *F. dibotrys* is an important crude drug which has been widely recorded in a series of Chinese medicine books, such as *New Compilation of Materia Medica, Supplements to Compendim of Materia Medica*. In China, the rhizome *F. dibotrys* is used as a folk herbal medicine for the treatment of lung disease, swelling and pain in throat and dysmenorrhea, inflammation, dysentery, and so on. In the Chinese Pharmacopoeia, *F. dibotrys* has been recorded to possess effects of heat-clearing and detoxicating, abscess and stasis removing.

*Fagopyrum dibotrys* is also an important part of many modern Chinese patent medicine formulations, such as Wei Mai Ning Capsule, Ji Zhi Tang Jiang, Fu Ping Jiao Nang, Jin Hua Ming Mu Wan, Chang Xin Jiao Nang, Jin Ci Can Jiu Zheng He Ji, etc. (Table 1). Thereinto, Wei Mai Ning Capsule which contains extract of *F. dibotrys* as the main raw material has been approved and clinical used as an alternative cancer treatment and is documented to increase the efficacy and reduce the toxicity of radiotherapy and chemotherapy. Ji Zhi Tang Jiang (consists of *Houttuyniae*, *F. dibotrys*, *Ilicis Chinensis Folium*, etc.) is a very famous over-the-counter drug which is effective in expectorating and relieving cough, and Fu Ping Jiao Nang (consists of *F. dibotrys*, *Viola yedoensis makino*, *Rhizoma curcumae*, etc.) is used in clinic for diseases such as pelvic inflammation and accessory inflammation.

**Table 1:** The ethnomedicinal uses of *Fagopyrum dibotrys*

Formulation	Main components	Ethnomedicinal uses
Wei Mai Ning Capsule	*F. dibotrys*	Promote blood circulation and remove blood stasis. Increase the effect and reduce the toxicity of cancer radiotherapy and chemotherapy
Ji Zhi Tang Jiang	*Houttuyniae*, *F. dibotrys*, *Ilicis Chinensis Folium*, *Asteris*, *Ephedrae*, *Peucedani*, *Aurantii Fructus*, *Glycyrrhizae*	Clear heat and transform phlegm; diffuse the lung and suppress cough
Fu Ping Jiao Nang	*F. dibotrys*, *Viola yedoensis makino*, *Rhizoma curcumae*, *Patrinia scabiosaefolia Fisch*, *Polygonum perfoliatum* L., *Sargentodoxa cuneata*, *Soldago decurrens Lour*	Remove toxicity for detumescence; clear heat for detumescence
Chang Xin Jiao Nang	*F. dibotrys*, *Hawthorn*, *F. tataricum*	Expel evil-wind and remove dampness; heat-clearing and detoxicating; eliminate stagnant blood
Jin Hua Ming Mu Wan	*Radix rehmanniae preparata*, *Semen cuscutae*, *Fructus lycii*, *Schisandra chinensis*, *Radix paeoniae alba*, *Polygonatum sibiricum red*, *Codonopsis pilosula*, *Rhizoma Chuanxiong*, *Chrysanthemum*, *Cassia obtusifolia*, *Semen plantaginis*, *Buddleja officinalis maxim*, *Endothelium corneum*, *F. dibotrys*, *Hawthorn*, *Cimicifuga foetida*	Reinforce liver and kidney; tonify kidney for improving eyesight
Jin Ci Can Jiu Zheng He Ji	*Rosa roxburghii*, *Sophora flavescens*, *F. dibotrys*	Nourish the stomach to improve the production of body fluid; improve the side effects of chemotherapy
Hong Jin Xiao Jie Nong Suo Wan	*Radix notoginseng*, *Cyperus rotundus*, *Dysosma versipellis*, *Armadillidium Vulgare*, *Black ant*, *Kadsura longipedunculata Finet et Gagn*, *Paederia scandens*, *Dahongpao*, *F. dibotrys*, *Radix Bupleuri*	Relieving the depressed liver; soften and resolve hard mass; promote blood circulation and remove blood stasis; reduce swelling and alleviate pain
Wu Jin Huo Xue Zhi Tong Pian	*Radix Paeoniae rubra*, *Viridifloric*, *F. dibotrys*	Promote blood circulation and remove blood stasis; remove obstruction in channels to relieve pain
Ge Shi Jiao Nang	*Puerariae*, *Astragalus*, *Herba epimedii Crneate lespedeza*, *F. dibotrys*, *Eucommia ulmoides*, *Rehmannia glutinosa*, *Scrophularia ningpoensis*, *Trichosanthin*, *Panax ginseng*	Supplement qi and nourish yin; promote the production of body fluid to quench thirst

## Phytochemicals

**Fig. 1:**
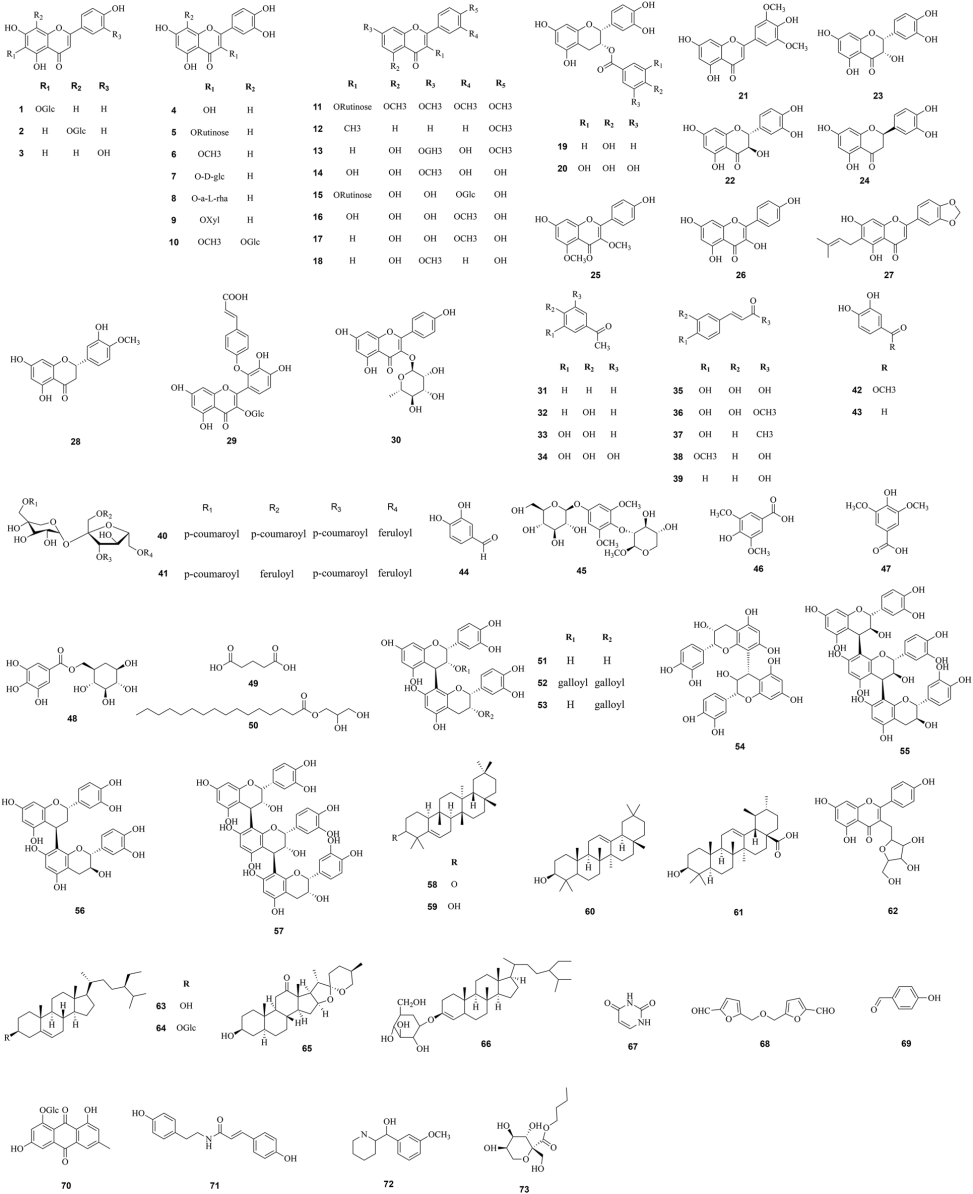
Chemical structures of the components isolated from *Fagopyrum dibotrys*

The phytochemicals distributed in *F. dibotrys* have been widely studied in the past decades. To date, a number of compounds with diverse structures have been isolated from *F. dibotrys*, primarily including flavonoids, phenolics, triterpenoids, tannins, steroids, fatty acid, etc. Essential volatile oils of *F. dibotrys* consist of aliphatic compounds, aromatic compounds, and sulfur-containing nitrogen compounds. Compounds that have been isolated and identified are listed in Table 2, and relative structures of these compounds are shown in Fig. 1.

**Table 2:** Phytochemistry of *Fagopyrum dibotrys*

No.	Compounds	No.	Compounds
**Flavonoids**		**Tannins**	
** 1**	Isovitexin	** 51**	Procyanidine B-2
** 2**	Vitexin	** 52**	3,3′-di-*O*-Galloyl-procyanidin B-2
** 3**	Luteolin	** 53**	3′-*O*-Galloyl-procyanidin B-2
** 4**	Quercetin	** 54**	Procyanidine B-4
** 5**	Rutin	** 55**	Procyanidine C-2
** 6**	3-Methylquercetin	** 56**	Procyanidin B-1
** 7**	Hyperin	** 57**	Procyanidine C-1
** 8**	Quercetin-3-*O*-α-l-rhamnoside	**Triterpenoids**	
** 9**	Quercetin-3-*O*-rhamnoside	** 58**	Glutinone
** 10**	3-Methyl-gossypetin 8-*O*-β-d-glucopyranoside	** 59**	Glutinol
** 11**	5,7,3′,4′-Tetrahydroxyflavan	** 60**	Olean-12-ene-3β,7β,15α,28-tetraol
** 12**	Pratol	** 61**	Ursolic acid
** 13**	Luteolin-7,4′-dimethyl ether	** 62**	Juglangenin A
** 14**	Rhamnetin	**Steroids**	
** 15**	Quercetin-3-*O*-rutinoside-3′-*O*-β-glucopyranoside	** 63**	β-Sitosterol
** 16**	Isorhamnetin	** 64**	Daucosterol
** 17**	Chrysoeriol	** 65**	Hecogenin
** 18**	Genkwanin	** 66**	β-Daucosterol
** 19**	(−)Epicatechin-3-*O*-p-hydroxybenzoate	**Other compounds**	
** 20**	3-Galloyl(−)-epicatechin	** 67**	Emodin
** 21**	Tricin	** 68**	5,5′-di-α-Furaldehy de dimethyl ether
** 22**	(+)-Catechin	** 69**	*p*-Hydroxy benzaldehyde
** 23**	(−)-Epicatechin	** 70**	Emodin-8-*O*-β-d-glucopyranoside
** 24**	Eriodictyol	** 71**	*N*-*trans*-Coumaroyl tyramine
** 25**	3,5-Dimethylquercetin	** 72**	(3-Methoxyphenyl)-2-piperidinemethanol
** 26**	Kaempferol	** 73**	*n*-butyl-β-d-Fructopyronoside
** 27**	3′,4′-Methylenedioxy-7-hydroxy-6-isopentenyl		
** 28**	Hesperitin		
** 29**	Quercetin-3-*O*-(2″-*O*-*p*-hydroxy-coumaroyl)-glucoside		
** 30**	Afzelin A		
**Phenolic**			
** 31**	Benzoic acid		
** 32**	*P*-Hydroxybenzoic acid		
** 33**	Protocatechuic acid		
** 34**	Gallic acid		
** 35**	Caffeic acid		
** 36**	Methyl caffeate acid		
** 37**	*trans*-*p*-Hydroxy cinnamic methyl ester		
** 38**	Ferulic acid		
** 39**	*P*-Coumaric acid		
** 40**	Diboside A		
** 41**	Lapathoside A		
** 42**	Protocatechuic acid methyl ester		
** 43**	3,4-Dihydroxy benzamide		
** 44**	3,4-Dihydroxy benzoic acid		
** 45**	1,3-Dimethoxy-2-*O*-b-xylo-pyranosyl-5-*O*-b-glucopyranosyl-benzene		
** 46**	3,5-Dimethoxy benzene carbonic acid-4-*O*-glu		
** 47**	Syringic acid		
** 48**	6-O-Galloyl-d-glucose		
** 49**	Succinic acid		
** 50**	Glycerol monopalmitate		

### Flavonoids

Flavonoids are naturally occurring polyphenolic compounds characterized by a carbon-based skeleton (C6–C3–C6) [13] and are widely presented in Chinese herbal medicines. Flavonoids have been shown to be the major active ingredients in *F. dibotrys*. The content of flavonoids in *F. dibotrys* is higher than that found in other buckwheat cultivars. To date, more than 30 flavonoids have been isolated from *F. dibotrys*, such as quercetin and rutin. In addition, catechins, (+)-catechin, and (−)-epicatechin, have also been isolated from *F. dibotrys*. These isolated flavonoids are listed in Table 2.

### Phenolics

Phenolics refer to nonpolymeric phytochemical compounds that contain at least one phenolic hydroxyl located on the same benzene ring [14]. Phenolics are widely distributed in plants and have a variety of physiological activities. To date, about 20 phenolics have been identified from *F. dibotrys*. The main phenolic compounds are derivatives, such as benzoic acid and 3,4-dihydroxy benzoic acid. In addition, phenolic compounds such as gallic acid, succinic acid, syringic acid, and ferulic acid can be used directly in various applications.

### Tannins

Tannins contain polyphenols and are widely reported to scavenge free radicals and prevent oxidation [15]. Seven tannins have been isolated from *F. dibotrys*. Among them, 3,3′-di-*O*-galloyl-procyanidin B-2 and 3′-*O*-galloyl-procyanidin B-2 are rich in hydroxyl groups.

### Triterpenoids

According to previous reports, a total of five triterpenoids have been extracted from *F. dibotrys*: glutinone, glutinol, 3α,21β-dihydroxy-olean-12-ene, olean-12-ene- 3β,7β,15α,28-tetraol, and ursolic acid.

### Steroids

Aside from the compounds mentioned above, several steroids have also been identified from *F. dibotrys*, mainly including 3α-hydroxy-urs-12,15-dien, Hecogenin, β-sitosterol, *N*-butanol-β-d-furan methylglycoside, *n*-butyl-β-d-fructopyronoside, β-daucosterol, and daucosterol.

### Volatile oils

**Fig. 2:**
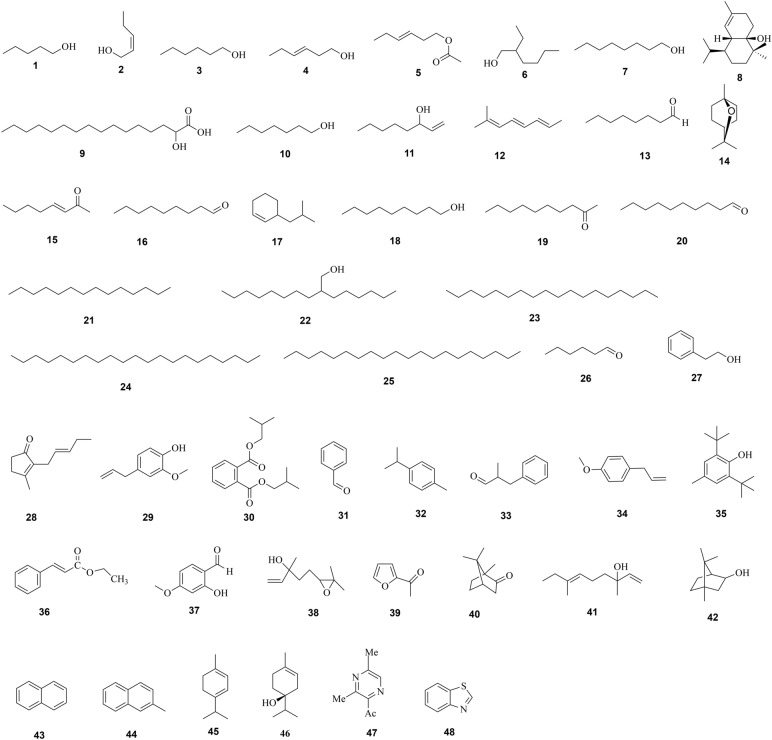
Chemical structures of the components isolated from the volatile oil of *Fagopyrum dibotrys*

Essential volatile oils consist of aliphatic compounds, aromatic compounds, terpenes (oxymonoterpenes, monoterpenes and oxygen sesquiterpenes), and sulfur-containing nitrogen compounds. To date, more than forty constituents have been isolated from *F. dibotrys*. Eugenol, α-terpineol, nonanal, (E)-3-Hexen-1-ol and ethyl cinnamate are the main components. In addition, different types of volatile oils derived from *F. dibotrys* have been shown to have anti-bacterial and anti-oxidant effects [16]. More detailed information on volatile oils can be found in Table 3, and their structures are listed in Fig. 2.

**Table 3:** Components isolated from the volatile oil of *Fagopyrum dibotrys*

No.	Compounds	No.	Compounds
**Aliphatic**		**Aromatic**	
** 1**	1-Pentanol	** 27**	Phenylethyl alcohol
** 2**	(Z)-2-Penten-1-ol	** 28**	(Z)-Jasmone
** 3**	1-Hexanol	** 29**	Eugenol
** 4**	(E)-3-Hexen-1-ol	** 30**	1,2-Benzenedicarboxylicacid,bis(2-methylpropyl) ester
** 5**	(E)-3-Hexen-1-yl acetate	** 31**	Benzaldehyde
** 6**	2-Ethyl-1-hexanol	** 32**	1-4-(1-Methylethyl)-benzene)
** 7**	1-Octanol	** 33**	2-Methyl-3-pheyl-propanal
** 8**	t-Muurolol	** 34**	1-Methoxy-4-(1-propenyl)-benzene
** 9**	Hexadecanoic acid	** 35**	Butylated hydroxytoluene
** 10**	1-Heptanol	** 36**	Ethyl cinnamate
** 11**	1-Octen-3-ol	** 37**	2-Hydroxy-*p*-anisaldehyde
** 12**	1,6-Dimethylhepta-1,3,5-triene	**Terpenes**	
** 13**	Octanal	** 38**	Linalool oxide
** 14**	Eucalyptol	** 39**	1-(2-Furanyl)-ethanone
** 15**	3-Octen-2-one	** 40**	Camphor
** 16**	Nonanal	** 41**	Isoborneo
** 17**	3-(2-Methylpropyl)-cyclohexene	** 42**	Borneol
** 18**	1-Nonanol	** 43**	Naphthalene
** 19**	2-Decanone	** 44**	2-Methyl-naphthalene
** 20**	Decanal	** 45**	α-Terpinene
** 21**	Tetradecane	** 46**	Terpinene-4-ol
** 22**	2-Hexyl-1-decanol	**Sulfur-containing nitrogen**	
** 23**	Octadecane	** 47**	1-(3,5-Dimethyl-2-pyrazinyl)-1-ethanone
** 24**	Heneicosane	** 48**	Benzothiazole
** 25**	Eicosane		
** 26**	Hexanal		

### Other compounds

In addition to the above components, *F. dibotrys* contains many other compounds, such as emodin, emodin-8-*O*-β-d-glucopyranoside, *N*-*trans*-coumaroyl tyramine, (3-methoxyphenyl)-2-piperidinemethanol, *n*-butyl-β-d-fructopyronoside and 5,5′-di-α-furaldehyde dimethyl ether. According to previous studies [17], *F. dibotrys* is also rich in protein, starch, fat, crude fiber, vitamins, and mineral elements. Table 2 contains information on these additional compounds.

## Pharmacological activities

**Fig. 3:**
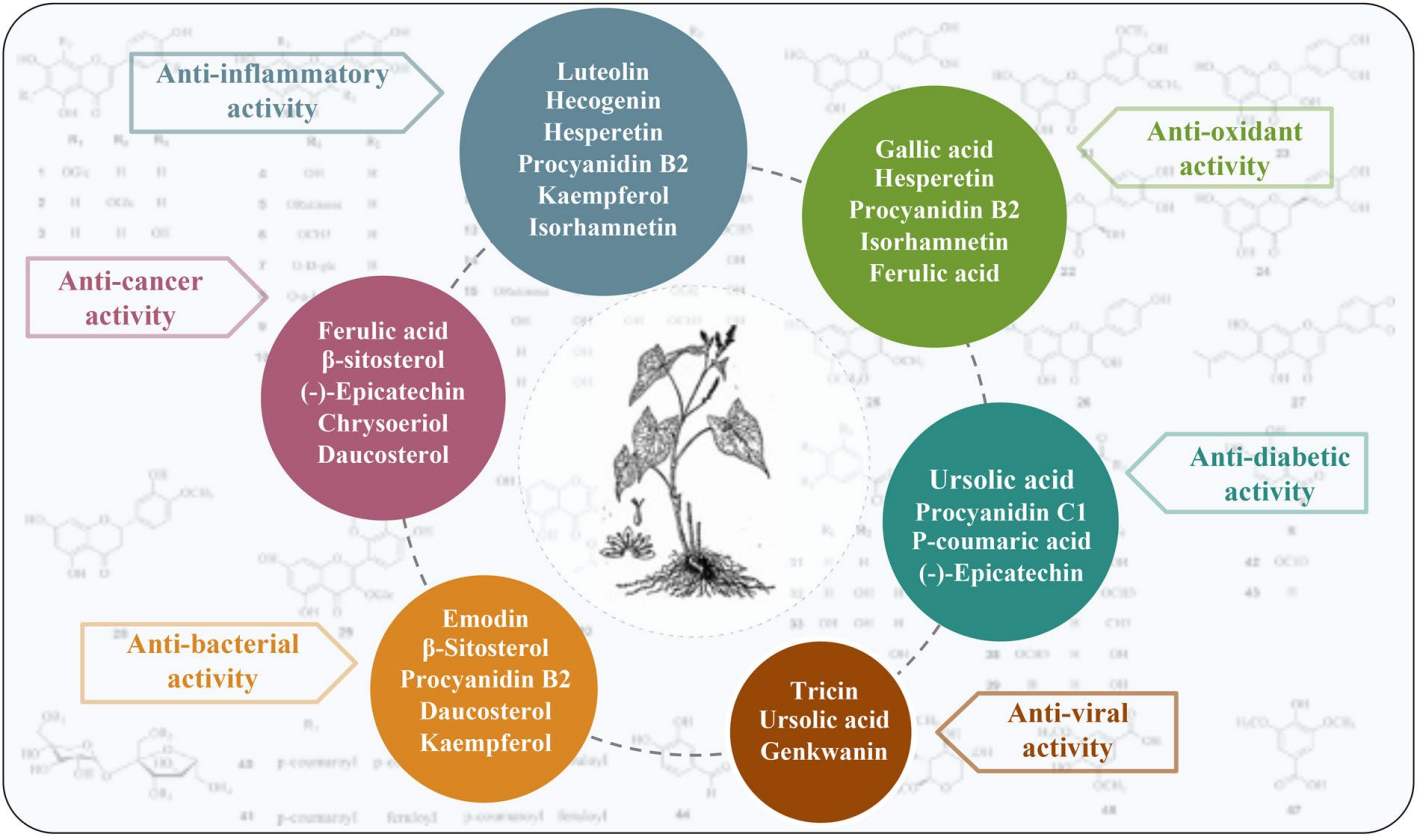
Pharmacological activities of *Fagopyrum dibotrys*

Due to its rich chemical composition, *F. dibotrys* has been reported to possess a variety of pharmacological activities, including anti-inflammatory, anti-cancer, anti-oxidant, anti-viral, anti-diabetic and anti-bacterial activities (Fig. 3). This review summarizes the various biological activities of *F. dibotrys* extracts and relative compounds (Table 4), which is conducive to further research on *F. dibotrys*.

**Table 4:** The pharmacological activities and mechanisms of the extracts and pure compounds from *Fagopyrum dibotrys*

Pharmacological activities	Components	Experimental models	Administration design	Effects
Anti-inflammatory	Ethanolic extract	FCA-induced AA rat model	40, 80 or 1600 mg/kg, 21 days (qd), i.g.	Decrease the plasma viscosity and reduce the production of IL-1 and TNF-a
	Procyanidin B1	LPS-induced THP-1	50, 100, 150 or 200 mg/L,18 h	Negative modulation Toll-like receptor-MyD88 signaling pathway
	Ethanolic extract	AA-induced irritable bowel syndrome rat model	6 or 24 g/kg, 14 days (qd), i.g.	Reduce intestinal inflammation via regulating tight junction proteins
	Ethanolic extract	FCA-induced SD rats	40, 80 or 160 mg/kg, 21 days (qd), i.g.	Reduce the production of IL-1 and TNF-a
	Luteolin	LPS-induced BALB/c mice	0.5 or 1 mg/kg, 2 h, i.p.	Inhibit HMGB1 and the production of TNF-a and NO
	Chrysoeriol	TPA-induced ear edema mouse model and LPS-stimulated RAW264.7 cells	0.5 or 1 mg/ear, 30 min, skin 10, 20 or 30 µM ,1 h	Inhibit JAK2/STA T3 and IκB/p65 NF-κB pathways
	Isorhamnetin	LPS-induced HGFs	10, 20 or 40 µM, 24 h	Inhibit LPS-induced inflammation in HGFs by activating Nrf2 pathway
	Tricin	LPS-induces hPBMCs	15 µM, 24 h	Anti-inflammatory via a mechanism involving the TLR4/NF-κB/STAT cascade
	Protocatechuic acid	RA-FLSs were obtained via collagenase digestion	5, 10, 20, or 40 µM, 24 or 48 h	Ameliorate RA-FLSs via inhibiting the NF-κB and Akt/mTOR signalling pathways
	Hecogenin	2,4-Dinitrofluorobenzenein-induced BALB/c mice	50, 75 or 100 mg/mice	Ameliorate inflammation by inhibiting TNF-a and IL-12
	Procyanidin B2	Paraquat-induced SD rats	25, 50 and 100 mg/kg, 3 days (qd), i.g.	Suppress the activation of NLRP3 inflammasome
Anti-cancer	Ethanolic extract	Ketamine-induced SD rats	6, 3, 1.5 g/kg 6 days (qd), i.g.	Decrease the excessive expression of TNF-α, ICAM-1, NF-κB p65 and MIP2M mRNA
	Extract	HeLa, DU145, H460, MCF-7, K562, HCT116, HepG2, U2OS, T98G cells	15, 30, 45, 60 or 120 µg/mL, 48, 72 or 96 h	Inhibit the proliferation of cells derived from certain organs
	Ethanolic extract	Hela cell	2, 10, 50, 250 g/mL, 12, 24, 48 or 72 h	Promote the expression of apoptotic Bax and inhibiting the expression of Bcl-2
	β-Sitosterol	MCF-7 cell	5, 10, 20 or 30 µM, 48 h	Preferentially binds with specific β-tubulin isotypes (βII and βIII)
	Rhamnetin	MCF-7 cell	5, 10, 15 or 25 µM, 24, 48 or 72 h	Induce apoptosis in MCF-7 cells via the miR‑34a/Notch‑1 pathway
	Isorhamnetin	PANC-1 cell	10–100 mol/L, 48 h	Decrease the phosphorylation levels of MEK and ERK in the Ras/MAPK pathway
	Genkwanin	HT-29 or sr-480	0.1, 1.0, 5.0 or 10.0 ng/mL, overnight	Inhibit cancer cells proliferation
	Afzelin	LNCaP or PC-3	0.1, 1.0 or 10.0 µg/mL, 72 h	Inhibition of LIM domain kinase 1 expression
Anti-oxidant	Crude aqueous acetone extract	DPPH	2-1000 g/mL, 30 min	DPPH radical scavenging
	Hesperidin	APAP-induced Wister rats	100 or 200 mg/kg, 14 days (qd), i.g.	Decrease caspase-3, caspase-9, NF-κB, iNOS, Kim-1 and increase Bcl-2
	Ferulic acid	ABTS and DPPH	1–10 µM, N/A	Enhance the scavenging of ABTS and DPPH radicals
	Procyanidin B2	HL-60	20 or 200 µM, 1 h	Exerts antioxidant by interacting with H_2_O_2_
Anti-bacterial	Ethanolic extract	Pneumococcus-induced KunMing mice	0.76, 0.38, 0.19, 0.098 or 0.049 g/kg,7 days (qd), i.g.	Prevent ethanol-induced acute liver injury via blocking CYP2El-mediated ethanol bioactivation and scavenging free radical
	Superfine powder *Fagopyrum dibotryis*	Gram-negative bacteria-induced KunMing mice	1.75, 1.25, 0.75 g/kg, 7 days (qd), i.g.	Against Gram-negative bacteria
	Emodin	TSB and TSA used for *H. parasuis* growth	32 or 64 µg/ mL, 24 h	Influence conformation of membrane protein
Anti-viral	Ethanolic extract	H3N2-induced chicken embryo	10, 5 or 2.5 mg/mL, 12 or 24 h	Inhibit viral proliferation
	Genkwanin	ASFV Ba71V	40 µM, 96 h	Reduce ASFV early and late proteins and viral DNA synthesis
	Tricin	H1N1pdm, H1N1, H3N2	3.3–30 µM, 8 h	Decrease the expression of viral protein and messenger RNA
	Ursolic acid	RV SA11	10, 20, 30, 40 µM, 24 h	Decrease VP6 and NSP2 viral proteins
	Procyanidin B1	Vesicular stomatitis and HCV-induced Huh-7	12.5, 25, 50 or 100 µM, 1 h	Suppress HCV RNA synthesis
	Procyanidin B2	FCV-F9 and MNV-1	1 or 0.5 mg/mL, 3, 6, 24 h	Causes structural changes of the P domain of VLPs, leading to HNoV inactivation
Anti-diabetic	Ethanolic extract	Streptozotocin-induced KunMing mice	50, 100, or 200 mg/kg, 6 weeks (qd), i.g.	Hypoglycemic via Crease TG, TC, LDL-C, MDA and HOMA-IR. Decrease HDL-C, SOD, CAT, GSH-Px and ISI
	Ursolic acid	Metabolic syndrome patient	150 mg/day, 12 weeks	Reduce body weight, BMI, waist circumference and fasting glucose, increase insulin sensitivity
	Procyanidin C1	ATCC CL173	1, 10 or 20 µM, 48 h	Act as a potential insulin action enhancer through the AKT-eNOS pathway in mature adipocytes
	Gallic acid and *P*-coumaric acid	STZ-induced *Rattus norvegicus*	20 mg/kg and 40 mg/kg, 6 weeks (qd), i.g.	Decrease the level of TNF-α and increased the levels of PPARγ mRNA and adiponectin

### Anti-inflammatory activity

Inflammation represents a defense mechanism of different organisms to various harmful stimuli [18]. Ethanol extract of *F. dibotrys* has been shown to exert anti-inflammatory effects in the treatment of colitis and arthritis. *F. dibotrys* extract can reduce lipopolysaccharide (LPS) levels, thereby inhibiting NF-κB p65 nuclear translocation and IκB phosphorylation and improving LPS-induced cell proliferation. At the same time, *F. dibotrys* extract can also reduce levels of pro-inflammatory cytokines and be used as a treatment for ulcerative colitis [4]. In cases of inflammatory bowel disease induced by dextran sodium sulfate, *F. dibotrys* can down-regulate tumor necrosis factor-α (TNF-α), as well interleukin (IL)-6, IL-1β and IL-10. By reducing expression of these inflammatory factors, *F. dibotrys* can thereby prevent and reduce inflammatory symptoms and damage [19]. In both in vivo and in vitro experiments, *F. dibotrys* has been shown to have a therapeutic effect on irritable bowel syndrome by up-regulating expression of claudin-1, claudin and ZO-1 tight junction proteins in colon epithelial cells, thereby reducing intestinal inflammation and enhancing mucosal epithelial function [20]. Shen et al. proved that *F. dibotrys* can inhibit experimental arthritis and reduce inflammation [21]. In experimental arthritis rats, *F. dibotrys* extract was shown to inhibit swelling and weight loss, reduce plasma viscosity, and significantly reduce production of proinflammatory cytokines IL-1 and TNF-α in serum. Treatment with *F. dibotrys* has also been shown to exert a protective effect on the lung tissue of rats with *Klebsiella* pneumonia [22].

The anti-inflammatory activity of *F. dibotrys* is primarily derived from the monomer compounds isolated from it. Previous in vitro and in vivo experiments have demonstrated that anti-inflammation is one of the main pharmacological effects of luteolin, and the main mechanism include regulation of transcription factors STAT3, NF-κB and AP-1 [23]. Isorhamnetin, another flavonoid compound isolated from *F. dibotrys*, has been proven to inhibit LPS-induced inflammation in human gingival fibroblasts by activating the Nrf2 signaling pathway [24]. Chrysoeriol can improve TPA-induced acute skin inflammation in mice, and thus also has an anti-inflammatory effect [25]. Tricin exerts anti-inflammatory effects through a mechanism involving TLR4/NF-κB/STA, which has protective effects on LPS-induced human peripheral blood mononuclear cells (hPBMCs) and carrageenan-induced rats [26]. Hesperetin and Kaempferol are also proved to modulate expression of pro-inflammatory cytokines and chemokines [27]. In the treatment of rheumatoid arthritis, protocatechuic acid can inhibit secretion of inflammatory cytokines, such as TNF-α, IL-1β and IL-6 [28]. Similarly, hecogenin can reduce production of TNF-α and IL-12 in Balb/c mice; when used in combination with flutica, it has a significant effect on skin inflammation and bronchitis [29]. In paraquat-induced acute lung injury in rats model, Procyanidin B2 was found to ameliorate oxidative stress, inhibit the expression of IL-1β and IL-18, and significantly reduce paraquat-induced activation of inflammatory factors in lung tissue [30]. Procyanidin B1 can negatively regulate the TLR-MyD88 signaling pathway and inhibit LPS-mediated inflammation [31]. Procyanidin can inhibit the activity of ERK1/2 and IKKβ to reduce production of LPS-induced production of reactive oxygen species, thereby exerting an anti-inflammatory effect [32].

### Anti-cancer activity

In the past decades, the anti-cancer activity of *F. dibotrys* has received more and more attention. Many studies have demonstrated that *F. dibotrys* and its components exhibit significant in vitro and in vivo inhibitory effects in various cancer types. *F. dibotrys* extract treatment could inhibit the growth of various cancer cells including lung cancer (H460), liver cancer (HepG2), leukocytes (K562), colon cancer (HCT116) and bone cancer (U2OS) [33]. *F. dibotrys* extract administration can inhibit the transplant ability of S180 sarcoma, Lewis lung cancer and U14 cervical cancer in mice models [24, 34]. Fr4 represents the fourth fraction isolated from *F. dibotrys* extract with more than 50% polyphenol content, it can down-regulate expression of matrix metalloproteinase (MMP)-9 and significantly inhibit the progression of Lewis lung cancer in mice [35].

Cancer prevention and treatment using combination therapy with Chinese medicines have been proposed as an effective strategy in clinic during the past years [36, 37]. Particularly, *F. dibotrys* extract in combination with other drugs have been documented to increase the efficacy and reduce the toxicity of radiotherapy and chemotherapy. Wei Mai Ning Capsule which contains extract of *F. dibotrys* as the main raw material has been approved for clinical cancer therapy. Combination of extracts from *Rosa roxburghii* Tratt and *F. dibotrys* significantly promoted apoptosis and inhibited growth of human esophageal squamous cell carcinoma CaEs-17, human gastric cancer SGC-7901 and lung cancer A549 cells [38]. The combination of daunorubicin and *F. dibotrys* extract has also been shown to have a synergistic effect.

The main components responsible for the anti-cancer activity of *F. dibotrys* have been investigated, as well as the underlying mechanisms. β-sitosterol (β-SITO), a compound found in *F. dibotrys*, has been demonstrated to decrease the viability of breast cancer MCF-7 cells, and immunofluorescence analysis confirmed the tubulin-targeted anticancer potential of β-SITO [39]. (−)-Epicatechin has been reported to suppress cancer cell proliferation through the MAP kinase pathway. (−)-Epicatechin treatment significantly improves the clone survival rate of normal human skin fibroblasts exposed to radiation, supporting its potential to be used both as a preventive agent and as an adjunct to chemotherapy and radiotherapy [40]. Murexin and Isovitexin have also been recorded to show anti-cancer activity in various cancer cells [41]. Rhamnetin treatment significantly promotes expression of P5 protein and miR-34a, subsequently inhibit the expression of Notch1 and suppress the proliferation of MCF-7 cells [42]. Isorhamnetin can down-regulate the expression of cyclin A to cause S-phase arrest and reduce the migration of advanced pancreatic cancer cells [43]. Genkwanin can significantly inhibite proliferation of human colorectal cancer HT-29 and SW-480 cells. Oral administration of genkwanin can improve the spleen and thymus indexes of the mice, as well as immune cytokine secretion, supporting genkwanin to be an effective compound for colorectal cancer [44]. Many studies have revealed the anti-cancer effect of hesperetin, which can down-regulate expression of glucose transporters to reduce the glucose uptake of cancer cells, thereby suppressing their proliferation. Hesperetin treatment can also inhibit cell migration by inhibiting TGF-β signaling [45]. In addition to anti-inflammatory effects, kaempferol also exhibits anti-cancer activity. Kaempferol treatment can induce G2/M phase cell cycle arrest and promote apoptosis in skin cancer and colon cancer cells [46]. Ursolic acid is a natural compound that exists widely in functional food including *F. dibotrys* and previous studies have shown that ursolic acid can block the G1/G2 cell cycle and induce breast cancer cell apoptosis [47]. Moreover, using the H-RS cell line, researchers found that procyanidin B2 can prevent the binding of NF-κB to DNA, inhibiting NF-κB-driven expression of genes including anti-apoptotic proteins [48].

### Anti-oxidant activity

*Fagopyrum dibotrys* contains a high concentration of polyphenols and flavonoids, which possess the ability to scavenge free radicals and superoxide anions. It has been documented to exhibit strong anti-oxidant capacity [49]. This anti-oxidant activity can be enhanced by the presence of two or three adjacent phenolic hydroxyl groups in the molecular formula [38, 50]. Many compounds identified in 60% of water acetone extracts were able to scavenge 1,1-diphenyl-2-picrylhydrazyl (DPPH) free radicals and showed significant anti-oxidant activity [50]. Compared with the positive control vitamin C, quercetin, dimethylquercetin and gallic acid found in *F. dibotrys* extract exhibited stronger anti-oxidant activity, and the ability of 6-*O*-galloyl-d-glucose to scavenge free radicals is even higher than that of vitamin C. Extracts of different parts of *F. dibotrys* have different anti-oxidant properties, with leaf extract showing the strongest anti-oxidant properties [51].

Hesperidin has a variety of biological activities, especially anti-inflammatory effects which is manifested in two main aspects: scavenging free radical activity and increasing the defense ability of anti-oxidant cells through the ERK/Nf2 signaling pathway [52]. Ferulic acid is widely used in cosmetics because of its anti-oxidant activity. In addition to scavenging free radicals, ferulic acid can also enhance the enzymatic activity of free radical scavengers and inhibitors [53]. Isorhamnetin treatment of a LPS-induced acute lung injury (ALI) mouse model indicated that isorhamnetin can inhibit activation of COX-2 and reduce the oxidative stress response induced by LPS, thereby preventing LPS-induced ALI [54]. Researchers also found that procyanidin B2 can inhibit H_2_O_2_-induced 8-oxo-7,8-dihydro-2 V-deoxyguanosine in human leukemia cell line HL-60. However, at high concentrations, it will increase the formation of 8-oxodG in HL-60 cells. Experiments with calf thymus DNA showed that procyanidin B2 reduced the M4PO-OH signal of H_2_O_2_ and Fe(II), but enhanced H_2_O_2_ and Cu(II) signals and induced DNA damage. Therefore, procyanidin B2 may interact with H_2_O_2_ and metal ions to exert both anti-oxidant and pro-oxidant effects [55].

### Anti-bacterial activity

The ethyl acetate extract of *F. dibotrys* has been proved to exhibit obvious anti-bacterial effect against *Streptococcus haemolyticus* B and pneumococcus in vitro. In vivo, the extract had an obvious protective effect on mice infected by pneumococcus. Based on the previous reports, the main anti-bacterial compounds are believed to be the phenolic acids, flavonoids and flavanol compounds [56]. The combined use of *F. dibotrys* and levofloxacin can improve the treatment efficacy of acute bacterial dysentery [57]. Studies have shown that *F. dibotrys* can be used to treat the lung tissue damage caused by *Klebsiella pneumoniae* by reducing expression of TNF-α, ICAM-1, NF-κB p65 and MIP-2 [22]. In another study, *F. dibotrys* was formulated into ultrafine powder and used at high, medium and low doses in mice infected with *Salmonella*. The results demonstrated that the ultrafine *F. dibotrys* powder had a protective effect on mice infected with *Salmonella* and suggested a dose-activity relationship [58].

Due to the abuse of antibiotics, antibiotic resistance has become a major threat to public health [59]. Discovery of natural products represents a compelling solution. β-SITO has been revealed to exhibit inhibitory effect on *Streptococcus pneumoniae*. β-SITO directly attacks the pneumolysin, the main virulence factor of pneumococcus, through two action sites (Thr459 and Leu460) to prevent cell lysis [44]. In studying the effect of β-SITO on mouse colitis, researchers found that it can significantly increase the expression of anti-microbial peptides in intestinal epithelial cells and reduce the survival rate of *Salmonella typhimurium* [60]. Emodin inhibits *Haemophilus parasui* by interacting with the cell membrane or proteins on the cell wall to change the conformation of membrane proteins [61]. Bacterial RecA stress stimulates DNA repair pathways, which are closely related to antibiotic resistance. P-coumaric acid is an effective RecA inhibitor, and has been shown to inhibit the biochemical activity driven by RecA and interfere with the DNA binding domain of the RecA protein [62].

### Anti-viral activity

Since the treatment of viral influenza with chemical drugs is prone to induce adverse reactions, such as drug resistance [63], more and more attention is now being paid to the use of traditional Chinese medicines as anti-viral treatments. Flavonoids from natural products have been widely reported to possess anti-viral potential [64]. Flavonoids also represent the main anti-viral components of *F. dibotrys*. Zhao et al. evaluated the anti-viral potential of *F. dibotrys* using both the hemagglutination test and a cell culture method, their results indicated *F. dibotrys* has anti-viral activity in vitro, and this activity is dependent on the dose of *F. dibotrys* flavonoids [65].

African swine fever virus (ASFV) is a serious threat to pig breeding due to the lack of effective treatment measures. In vitro experiments have shown that genkwanin can reduce protein levels and DNA synthesis in both early and late stages of ASFV, in addition to inhibiting the ASFV Ba71V strain in Vero cells [66]. Tricin has been proved to exhibit inhibitory effect against influenza virus by reducing expression of hemagglutinin and matrix protein in influenza virus-infected cells, as well as reducing the expression of HA and M messenger RNA. In addition, tricin has a positive effect on the body weight and survival rate of infected mice in a mouse model of influenza virus infection [67]. Recent study reported that ursolic acid has the potential to resist rotavirus, hindering early virus replication [68]. Procyanidin B1 was documented to be a new therapeutic agent for hepatitis virus C (HCV) treatment. It was found to inhibit the synthesis of HCV RNA in a dose-dependent manner, thereby preventing vesicular stomatitis virus and showing an inhibitory effect on HCV pseudo virus-infected Huh-7 cells [69]. The feline calicivirus (FCV)-F9 and murine norovirus (MNV)-1, both of which are alternatives to human norovirus (HNoV), were selected to test the anti-viral activity of procyanidin B2. It was found that procyanidin B2 can reduce the concentrations of both FCV-F9 and MNV-1 to undetectable levels. In addition, protocyanidin B2 was shown to bind directly to the main capsid structure of HNoV, namely the P domain, thus significantly changing the tertiary structure of the virus-like particles [70].

### Anti-diabetic activity

Previous studies indicated that reactive oxygen radicals and their oxidation reactions can damage islet β cells, leading to diabetes [71]. *F. dibotrys* extract is rich in amino acids and vitamins which can scavenge free radicals, reducing damage to the body and achieve anti-diabetic effects. Treatment with 50–200 mg/kg *F. dibotrys* flavonoids is effective in type 2 diabetic mice. In T2DM mice, this treatment can reduce blood glucose and insulin levels, increase body weight and regulate blood lipid metabolism and oxidative stress [72]. In vitro studies have revealed that ursolic acid can inhibit the related protein tyrosine phosphatase 1B, which leads to down-regulation of insulin receptors. Controlled clinical trials in patients with metabolic syndrome indicated that, compared to the placebo group, patients treated with oral ursolic acid showed relieved symptoms, increased insulin sensitivity and reduced concentration of fasting blood glucose [73]. Procyanidin C1 can improve differentiation of 3T3-L1 adipocytes and activate the AKT-eNOS pathway, thereby promoting insulin-induced glucose uptake in cells and increasing insulin sensitivity [74]. Moreover, in diabetic rats, *P*-coumaric administration led to a significant reduction in the levels of glucose and glycosylated hemoglobin, as well as induced significant increases in insulin and body weight. Additional anti-diabetic mechanisms of *P*-coumaric acid include reduced levels of TNF-α and increased levels of adipocytokines and PPARγ [75].

### Other activities

In addition to the above pharmacological activities, *F. dibotrys* also has several other activities. *F. dibotrys* contains a lot of polyphenolic compounds and has obvious anti-tussive and expectorant effects [76]. It also has a neuroprotective effect and can cross the blood-brain barrier to penetrate and accumulate in the brain. Therefore, *F. dibotrys* has a therapeutic effect on Alzheimer’s disease (AD) [77]. After intragastric administration of *F. dibotrys* flakes to rats, the collected serum can inhibit the contraction of guinea pig ileum in vitro [78]. In addition, *F. dibotrys* has an anti-mutagenic effect on mutations of TA98 and TA100 strains induced by orthodoxin and methanesulfonate [79].

*Fagopyrum dibotrys* extract has therapeutic potential for neurodegenerative diseases, and vitexin is suggested to be one of the main active compounds. Vitexin can increase neuroprotective factors and counteract the induction of neurodegeneration. Ferulic acid is also considered to be a promising treatment for AD [80]. Ferulic acid can reduce the oxidative stress response in AD and protect the brain from Aβ neurotoxicity [81]. The neuroprotective effects of procyanidin B2 evaluated in rat cerebellar granule neurons, the results indicated that procyanidin B2 exhibited neuroprotection effect by scavenging reactive oxygen and nitrogen [82]. Procyanidin C1 has been proved to act on glutamate-induced HT22 cells to significantly reduce cell death. This compound also possesses free radical scavenging activity, inhibiting the accumulation of reactive oxygen species and protein carbonylation in cells induced by glutamate, thus exhibits neuroprotective effect on HT22 cytotoxicity induced by glutamate [83].

## Functional genes

At present, research into medicinal plants primarily focuses on extraction and separation technology, effective ingredient analysis and assessment of pharmacological activity [78]. With increasing clinical demand for medicinal plants and the development of molecular biology techniques, research into functional genes related to the synthesis of active pharmaceutical ingredients has attracted significant interest. The research into functional genes of medicinal plants mainly emphasizes the investigation of the genes encoding key enzymes that play an important role in regulating the synthesis of secondary metabolites [84]. Secondary metabolites are produced by medicinal plants that have adapted to the environment in order to survive, and are divided into phenols, alkaloids, terpenes, etc. [85]. Previously, the functional genes of *F. dibotrys* have been studied, the genes related with the secondary metabolites of *F. dibotrys* have been discovered as well as the relevant metabolic pathways. Relative studies play an important role in research into the active ingredients of *F. dibotrys*. *F. dibotrys* contains many flavonoids, and its biological activity and synthetic pathways have received extensive attention. At present, the research into functional genes of *F. dibotrys* is primarily focused on flavonoids.

Anthocyanins are flavonoids, and the anthocyanin synthase (ANS) gene is critical to the anthocyanin synthesis pathway in golden buckwheat. The anthocyanin synthase (FdANS) gene was obtained from *F. dibotrys* for the first time using homologous cloning technology. Bioinformatics showed that the protein encoded by the FdANS gene was homologous to the ANS genes of other medicinal plants. The expression levels of FdANS in different tissues were analyzed; the results showed that the level of FdANS was flower > leaf > stem > root, but the amount of anthocyanin was flower > leaf > stem > root. The amount of penicillin is correlated [86]. The rhizome of *F. dibotrys* contains a lot of procyanidins (PAs). A radiation-induced mutant (RM_R) obtained by Chen et al. [3] contained higher procyanidins than wild-type (CK_R). RNA-seq was used to compare rhizome transcripts between the two strains. This analysis involved a total of 53,540 genes, of which 29,901 were annotated and analyzed. This analysis found that some of the single genes encoding PAs biosynthetic enzymes are different between RM_R and CK_R, with 501 unique sequences encoding the differential expression of single genes encoding TFs between RM_R and CK_R samples. Accumulation of cytochrome P450s in flavonoids, detected by qRT-PCR, showed the expression of twelve key genes related to flavonoid biosynthesis and was consistent with the RNA-seq results. Therefore, radiation increased the expression of PA synthesis-related genes, and accumulation made the PA content in RM_R higher than in the buckwheat rhizome of CK_R.

Dihydroflavonol 4-reductase (DFR) is a key enzyme for procyanidin synthesis. Using the RACE method combined with cDNA library screening, the DFR (FdDFR) gene was cloned from *F. dibotrys*. The DFR gene in the *F. dibotrys* genome is a small family of 1 to 2 genes. FdDFR1 is a single copy gene and is homologous to DFR genes of other medicinal plants [87]. A mixture of procyanidin-condensed tannin is one example of a secondary metabolite of flavonoids [34]. The leucoanthocyantin reductase (LAR) gene encodes a key enzyme in the synthesis of flavonoids. It was obtained from *F. dibotrys* by PCR and RACE. By using real-time fluorescence quantitative PCR technology, researchers found that the FdLAR gene was related with the accumulation of secondary metabolites of buckwheat flavonoids [88]. In addition, the chalcone isomerase (CHI) gene also plays an important role in the *F. dibotrys* flavonoid synthesis pathway [34]. The cDNA sequence of the CHI (FdCHI) gene was obtained from *F. dibotrys* by using homologous cloning technology. Bioinformatic analysis showed that the protein encoded by the FdCHI gene was homologous to the CHI genes of other medicinal plants, and FdCHI was expressed in different tissues of *F. dibotrys*. The results showed that expression of FdCHI was flower > root > leaf > stem, while total flavonoid content was flower > leaf > stem > root. This suggests that expression of FdCHI is related to the total flavonoid content in flowers, leaves and stems, but is less relevant in the root [89].

Rutin is another secondary metabolite in *F. dibotrys*. Rutin belongs to the flavonoid family, and its synthesis is inseparable from that of phenylalanine ammonia lyase (PAL). Through homologous cloning and RT-PCR technology, the DNA sequence of the *F. dibotrys* PAL gene was measured to be 2583 bp. The cDNA sequence contained an open reading frame of 2169 bp, encoding 722 amino acids. After synthesis, the acid was converted to cinnamic acid [65]. The specific characteristics of the PAL gene sequence can be used for the genetic improvement of golden buckwheat [90]. Flavanol synthase (FLS) enzyme and dihydroflavonol form quercetin through an oxidation reaction. It has been reported that the FLS gene is encoded by multiple gene copies, and the same copy can be found in different medicinal plants [91, 92]. Using homologous cloning technology, the FLS (FdFLS) gene was cloned and the cDNA sequence of FdFLS in *F. dibotrys* was determined [93].

Many transcription factors also play important roles in the *F. dibotrys* flavonoid synthesis pathway. Some studies have used RACE combined with a cDNA library screening method to clone the transcription factor gene MYBP1 (FdMYBP1) from *F. dibotrys*. Through southern hybridization and bioinformatics analysis, researchers revealed that FdMYBP1 gene exists in one to two copies, equal to MYB based on structural characteristics of the source gene, it is believed that FdMYBP1 plays a vital role in flavonoid metabolism [94].

## Discussion and future perspectives

*Fagopyrum dibotrys* is a famous edible herbal medicine widely used in traditional Chinese medicine. In China, *F. dibotrys* has been used for a long time to treat diseases such as pneumonia and tonsil swelling. As a traditional Chinese medicine, *F. dibotrys* is an important component of many Chinese medicine formulas, including Wei Mai Ning Capsule, Ji Zhi Tang Jiang, and Jin Hua Ming Mu Wan. To date, more than 70 chemical components have been isolated from *F. dibotrys*, together with nearly 50 components from the volatile oil. However, investigation on the whole chemical components of *F. dibotrys* is far from finish up completely. With the development of modern science and technologies in rapid and efficient separation and identification of complex chemical compounds from natural products [95], it is expected that more unknown compounds will be separated and identified from *F. dibotrys* using technologies such as multi-dimensional liquid phase chromatography combined with mass spectrometry. Better understanding on phytochemistry will undoubtedly promote the further investigation of golden buckwheat.

Previous research on the pharmacological activity of *F. dibotrys* has characterized its multiple effects which partially support its traditional uses. For example, a series of pharmacological studies focused on the anti-inflammatory activity of *F. dibotrys* and have proved its therapeutic potential in lung abscesses, irritable bowel syndrome, etc. The extracts of *F. dibotrys* and isolated compounds including hecogenin, luteolin, isorhamnetin and chrysoeriol have been demonstrated to suppress secretion of inflammatory cytokines and inhibit related pathways in relevant in vitro and in vivo models. Besides, according to the ethnomedicinal uses in China, Jin Ci Can Jiu Zheng He Ji, which is composed of *Rosa roxburghii*, *Sophora flavescens* and *F. dibotrys*, has been clinically used in adjuvant therapy for radiotherapy and chemotherapy. Accordingly, modern pharmacological studies demonstrated that the extracts and monomers of *F. dibotrys* exhibited anti-cancer activities in various cancer types in vitro and in vivo. Furthermore, Wei Mai Ning Capsule has been clinical used to increase the efficacy and reduce the toxicity of radiotherapy and chemotherapy. However, the main components responsible for the anti-cancer potential of *F. dibotrys* are not fully clarified, as well as the underlying molecular mechanisms. With the widespread application of targeted therapy and immunotherapy in clinical cancer treatment, the combination effects of *F. dibotrys*-related products with relative therapeutic strategies also worth further evaluation.

Previously, the anti-cancer and anti-oxidant activities of *F. dibotrys* have attracted most of the attention, while investigation on the pharmacological activities that supporting the folk medicinal usage of *F. dibotrys* are still insufficient. For example, *F. dibotrys* has been documented as an important Chinese medicine for its therapeutic effect against pulmonary abscess and pneumonia. However, the potential mechanisms and targets are still unclear. With the development of traditional Chinese medicine and the strengthening of the world’s understanding of traditional Chinese medicine, research on the anti-inflammation, anti-viral and immune-promoting effects of *F. dibotrys* should be synchronously promoted, and much more work is still needed. Previously, *F. dibotrys* extracts or monomers are mostly used in the *in vivo* and *in vitro* pharmacological studies. Studies have proved that *F. dibotrys* has a certain effect on pneumonia, enteritis, diabetes, and other diseases. However, in some of the pharmacological studies, the in vivo experimental dose is much too high, and some in vitro experimental administration time is very long, whether these experimental designs are reasonable should be taken into consideration in the future studies.

With developments in biochemistry, people are paying more and more attention to the secondary metabolites when researching medicinal plants. It has been documented that the contents of secondary metabolites in medicinal plants are related with the expression of different functional genes. Therefore, it is critical to start from functional genes to elucidate the biosynthetic pathways and regulatory mechanisms of active ingredients involved in the regulation of secondary metabolites and structure-encoding genes of key enzyme systems [96]. Previously, research into the secondary metabolites of *F. dibotrys* is mainly concentrated on flavonoids. In subsequent endeavors, research into other secondary metabolites including the polyphenols should be strengthened in order to further clarify the synthetic pathways and regulatory mechanisms of the effective ingredients of *F. dibotrys*.

In addition to the aforementioned phytochemical and pharmacological studies, *F. dibotrys* has also received significant attention because of its high nutritional and medicinal value. *F. dibotrys* seeds are often used as coarse grains and can be made into tea. *F. dibotrys* leaves can be eaten directly as vegetables [97]. *F. dibotrys* can be processed into highly nutritious feed or feed additives, and green straw can be used as pasture. Modern food processing technology and equipment may help further development of *F. dibotrys* related healthy products. More importantly, protection of the wild resources and development of cultivation techniques and genetic analysis will help better development and utilization of this medicinal economic plant.

## Conclusions

As a famous edible herbal medicine widely used in traditional Chinese medicine, *F. dibotrys* has attracted increasing attention in the past decades. To date, more than 100 compounds have been isolated and identified from *F. dibotrys*, mainly including flavonoids, phenols, steroids, and fatty acids, etc. Pharmacological studies have shown that *F. dibotrys* possesses anti-inflammatory, anti-cancer, anti-oxidant, anti-bacterial and anti-diabetic activities, which partially support the ethnomedicinal uses of *F. dibotrys*. Flavonoids and phenolic compounds are considered to be the main active ingredients of *F. dibotrys*. Additional studies on functional genes have led to a better understanding on the metabolic pathways and regulatory factors related to flavonoid active ingredients in *F. dibotrys*. Our study provides a comprehensive review regarding the research advances on the phytochemistry and pharmacology of *F. dibotrys*, as well as the functional genes, aiming to promote further research on *F. dibotrys*.

## Data Availability

Not applicable.

## Abbreviations

*F. dibotrys*: *Fagopyrum dibotrys*
*F. esculentum*: *Fagopyrum esculentum*
*F. tataricum*: *Fagopyrum tataricum*
LPS: Lipopolysaccharide
TNF-α: Tumor necrosis factor-α
IL: Interleukin
hPBMCs: Human peripheral blood mononuclear cells
ALI: Acute lung injury
DPPH: 1,1-Diphenyl-2-picrylhydrazyl
β-SITO: β-Sitosterol
ASFV: African swine fever virus
HCV: Hepatitis virus C
FCV: Feline calicivirus
MNV: Murine norovirus
HNoV: Human norovirus
AD: Alzheimer’s disease
MMP: Metalloproteinase
ANS: Anthocyanin synthase
DFR: Dihydroflavonol 4-reductase
LAR: Leucoanthocyantin reductase
CHI: C halcone isomerase
PAL: Phenylalanine ammonia lyase
